# Small but mighty: old and new parvoviruses of veterinary significance

**DOI:** 10.1186/s12985-021-01677-y

**Published:** 2021-10-24

**Authors:** Mason C. Jager, Joy E. Tomlinson, Robert A. Lopez-Astacio, Colin R. Parrish, Gerlinde R. Van de Walle

**Affiliations:** Baker Institute for Animal Health, College of Veterinary Medicine, Cornell University, Ithaca, NY 14853 USA

**Keywords:** Pathogenicity, Animal parvoviruses, Viral therapeutics, Viral metagenomics, Amdoparvovirus, Copiparvovirus, Chaphamaparvovirus

## Abstract

In line with the Latin expression “*sed parva forti”* meaning “small but mighty,” the family *Parvoviridae* contains many of the smallest known viruses, some of which result in fatal or debilitating infections. In recent years, advances in metagenomic viral discovery techniques have dramatically increased the identification of novel parvoviruses in both diseased and healthy individuals. While some of these discoveries have solved etiologic mysteries of well-described diseases in animals, many of the newly discovered parvoviruses appear to cause mild or no disease, or disease associations remain to be established. With the increased use of animal parvoviruses as vectors for gene therapy and oncolytic treatments in humans, it becomes all the more important to understand the diversity, pathogenic potential, and evolution of this diverse family of viruses. In this review, we discuss parvoviruses infecting vertebrate animals, with a special focus on pathogens of veterinary significance and viruses discovered within the last four years.

## Background

**Fig. 1:**
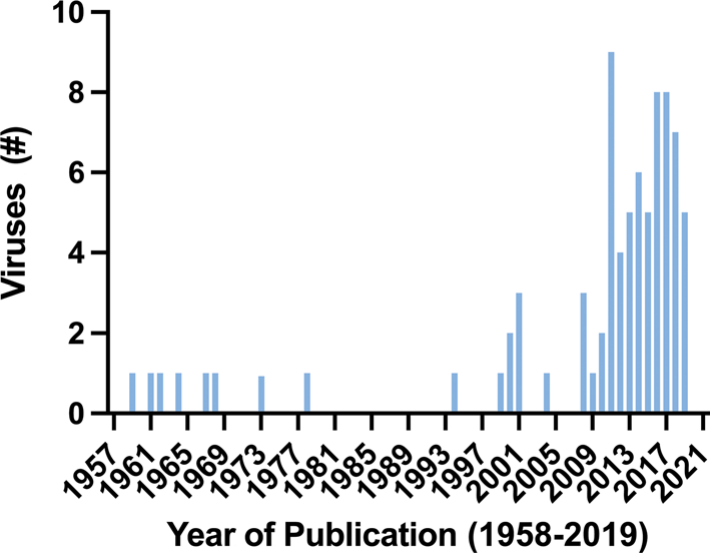
Number of animal parvoviruses discovery by year. Graph showing the number of new ICTV-recognized non-human, vertebrate animal parvoviruses [20] discovered between 1958 and 2019. Viruses discovered since 2019 have not been consistently added to the ICTV taxonomy and are, thus, excluded. Note the marked increase in viral discovery in the last 20 years due to the use of metagenomics and high-throughput sequencing

While diseases caused by viruses in the Family *Parvoviridae* have been known since the early twentieth century, the properties of these viruses were only revealed in the 1960s. High-throughput sequencing and new metagenomic analytical methods have greatly increased the number of new parvoviruses discovered in animals in recent years (Fig. 1, Table 1). For example, a recent DNA-sequencing virome study using feces collected from Australian ducks identified and characterized 46 different parvoviruses belonging to three different genera [1]. However, many of the recently discovered viruses are poorly understood beyond their DNA sequence. An added challenge in host assignment of novel viruses is that viral DNA detected in feces could originate from the animal or from its diet, as seen with the initial identification of tilapia parvovirus in the feces of a crocodile [2]. Finding viral DNA or virus in the tissues or blood of an animal provides greater certainty of the source of DNA.

**Table 1:** Summary of vertebrate animal parvoviruses by genus and species. Vertebrate animal parvoviruses from the ten genera of subfamily *Parvovirinae* and the genus *Chaphamaparvovirus* of subfamily *Hamaparvovirinae* are listed alphabetically by species, as proposed by the ICTV [20]

Genus	Species	Virus name	Abbrev	Tissue source	Age affected	References
*Amdoparvovirus*	*Carnivore amdoparvovirus 1*	Aleutian mink disease parvovirus	ADV	Tissue	Young/adult	[44]
	*Carnivore amdoparvovirus 2*	Gray fox parvovirus	GFAV	S, Lu	Unknown	[98]
	*Carnivore amdoparvovirus 3*	Racoon dog and fox amdoparvovirus	RFAV	S, K, MLN, blood	Young	[88]
	*Carnivore amdoparvovirus 4*	Skunk amdoparvovirus	SKAV	Tissue	Young/adult	[89]
	*Carnivore amdoparvovirus 5*	Red panda amdoparvovirus	RpAPV	S, Li, Lu, K, SI, feces	Unknown	[10]
	NA	*Labrador amdoparvovirus 1*	LaAV-1	S, LN, muscle	Unknown	[74]
	NA	*Labrador amdoparvovirus 2*	LaAV-2	S, LN, muscle	Unknown	[74]
*Artiparvovirus*	*Chiropteran artiparvovirus 1*	Artibeus jamaicensis parvovirus	Aj-BtPV-1	Blood	Unknown	[99]
*Aveparvovirus*	*Columbid aveparvovirus 1*	Pigeon parvovirus 1	PiPV1	Feces	Unknown	[125]
	*Galliform aveparvovirus 1*	Turkey parvovirus Chicken parvovirus	TuPV ChPV	I	Unknown	[346]
	*Gruiform aveparvovirus 1*	Red-crowned crane parvovirus	RcPV	Feces	Unknown	[127]
*Bocaparvovirus*	*Carnivore bocaparvovirus 1*	Minute virus of canines	MVC	Feces	Young	[133]
	*Carnivore bocaparvovirus 2*	Canine bocavirus 2	CBoV2	Respiratory	Unknown	[152]
	NA	Canine bocavirus 3	CBoV3	Li	Unknown	[155]
	*Carnivore bocaparvovirus 3*	Feline bocavirus 1	FBoV1	Feces, blood, K, nasal swabs	Unknown	144[]
	*Carnivore bocaparvovirus 4*	Feline bocaparvovirus 2	FboV2	Feces	Unknown	[145]
	*Carnivore bocaparvovirus 5*	Feline bocaparvovirus 3	FBoV3	Feces	Unknown	[146]
	*Carnivore bocaparvovirus 6*	Mink bocavirus 1	MiBoV1	Feces	Unknown	[167]
	*Chiopteran bocaparvovirus 1*	Myotis myotis (bat) bocavirus 1	BtBoV1	Pharyngeal and anal swabs	Unknown	[161]
	*Chiopteran bocaparvovirus 2*	Bat bocavirus WM40	BtBoVwm40	Tissue	Unknown	[162]
	*Chiopteran bocaparvovirus 3*	Bat bocavirus XM30	BtBoVxm30	Tissue	Unknown	[162]
	*Chiopteran bocaparvovirus 4*	Miniopterus schreibersii bat bocavirus	BtBoV2	S, Lu, I	Unknown	[163]
	*Chiopteran bocaparvovirus 5*	Rousettus leschenaultii bocaparvovirus 1	RlBoV	S, Li, I	Unknown	[164]
	*Lagomorph bocaparvovirus 1*	Rabbit bocaparvovirus	RBoV	Feces	Unknown	[168]
	*Pinniped bocaparvovirus 1*	California sea lion bocavirus 1	CslBoV1	Feces	Unknown	[169]
	*Pinniped bocaparvovirus 2*	California sea lion bocavirus 3	CslBoV3	Feces	Unknown	[169]
	*Primate bocaparvovirus 1*	Human bocavirus 1 and 3	HBoV1, 3	Respiratory	Young	[128]
	*Primate bocaparvovirus 2*	Human bocavirus 2 and 4	HBoV2, 4	Stool	Unknown	[347]
	*Primate bocaparvovirus 3*	Macca mulatta bocaparvovirus	MmBoV	Feces	Unknown	[348]
	*Rodent bocaparvovirus 1*	Rat bocavirus	RBoV	Lu, I, S, K	Unknown	[164]
	*Rodent bocaparvovirus 2*	Murine bocavirus	MuBoV	Feces	Unknown	[223]
	*Ungulate bocaparvovirus 1*	Bovine parvovirus 1	BPV1	I	Young	[140]
	*Ungulate bocaparvovirus 2*	Porcine bocavirus 1	PBoV1	LN	Unknown	[156]
	*Ungulate bocaparvovirus 3*	Porcine bocavirus SX	PBoVsx	Serum	Unknown	[349]
	*Ungulate bocaparvovirus 4*	Porcine bocavirus H18	PBoVh18	Feces	Unknown	[159]
	*Ungulate bocaparvovirus 5*	Porcine bocavirus 3	PBoV3	Feces	Unknown	[350]
	*Ungulate bocaparvovirus 6*	Bovine bocaparvovirus 2	BBoV2	Nasal swab	Unknown	[143]
	*Ungulate bocaparvovirus 7*	Dromedary camel bocaparvovirus 1	DBoV1	Feces	Unknown	[165]
	*Ungulate bocaparvovirus 8*	Dromedary camel bocaparvovirus 2	DBoV2	Feces	Unknown	[165]
	*Ungulate bocaparvovirus 9*	Vicugna pacos bocaparvovirus	VpBoV	Feces	Unknown	[351]
*Copiparvovirus*	*Pinniped copiparvovirus 1*	Sesavirus	SesaV	Feces	Unknown	[199]
	*Ungulate copiparvovirus 1*	Bovine parvovirus 2	BPV2	Serum	Unknown	[180]
	*Ungulate copiparvovirus 2*	Porcine parvovirus 4	PPV4	Lu lavage	Unknown	[170]
	*Ungulate copiparvovirus 3*	Roe deer copiparvovirus	RdPV	*Ixodes* tick	Unknown	[198]
	*Ungulate copiparvovirus 4*	Porcine parvovirus 6	PPV6	Fetus	Unknown	[194]
	*Ungulate copiparvovirus 5*	Bosavirus	BosaV	Serum	Unknown	[181]
	*Ungulate copiparvovirus 6*	Equine parvovirus-hepatitis	EqPV-H	Li, serum	Adult	[3]
	NA	Equine parvovirus-CSF	EqPV-CSF	CSF	Unknown	[196]
	NA	Equine copiparvovirus	EqCoPV	Plasma	Unknown	[178]
	NA	Sheep copiparvovirus	Sheep PV	Serum	Unknown	[200]
	*Adeno-associated dependoparvovirus A*	Adeno-associated virus 1, 2, 3, 4	AAV1-4	Cell culture	Unknown	[202]
	*Adeno-associated dependoparvovirus B*	Adeno-associated virus 5	AAV5	Cell culture	Unknown	[352]
*Dependoparvovirus*	*Anseriform dependoparvovirus 1*	Goose parvovirus Muscovy duck parvovirus Novel goose parvovirus	GPV MDPV nGPV	Tissue Tissue Li, S, heart	Young Young Young	[204] [209] [353]
	*Avian dependoparvovirus 1*	Avian adeno-associated virus	AAAV	Respiratory	Unknown	[215]
	*Carnivore dependoparvovirus 1*	Feline dependoparvovirus	FdPV	Feces	Unknown	[151]
	*Chiropteran dependoparvovirus 1*	Bat adeno-associated virus	BtAAV	Feces	Unknown	[219]
	*Pinniped dependoparvovirus 1*	California sea lion adeno-associated virus 1	CslAAV1	Feces	Unknown	[169]
	*Rodent dependoparvovirus 1*	Murine adeno-associated virus 1	MAAV1	Feces	Unknown	[223]
	*Rodent dependoparvovirus 2*	Murine adeno-associated virus 2	MAAV2	Feces	Unknown	[223]
	*Squamate dependoparvovirus 1*	Snake adeno-associated virus	SAAV	Heart, S, Li, K	Unknown	[224]
	*Squamate dependoparvovirus 2*	Bearded dragon parvovirus	BDPV	Lu, Li, I, K, gonads	Unknown	[227]
*Erythroparvovirus*	*Pinniped erythroparvovirus 1*	Seal parvovirus	SePV	Brain	Unknown	[235]
	*Primate erythroparvovirus 1*	Human parvovirus B19	B19V	Serum	Young/Adult	[354]
	*Primate erythroparvovirus 2*	Simian parvovirus	SPV	Serum	Young/Adult	[53]
	*Primate erythroparvovirus 3*	Rhesus macaque parvovirus	RmPV	Serum	Unknown	[232]
	*Primate erythroparvovirus 4*	Pig-tailed macaque parvovirus	PmPV	Serum	Unknown	[232]
	*Rodent erythroparvovirus 1*	Chipmunk parvovirus	ChpPV	Serum	Unknown	[233]
	*Ungulate erythroparvovirus 1*	Bovine parvovirus 3	BPV3	Serum	Unknown	[180]
*Loriparvovirus*	*Primate loriparvovirus 1*	Slow loris parvovirus 1	Sl.L-PV-1	I, Li, K, Lu, Serum	Unknown	[237]
*Protoparvovirus*	*Carnivore protoparvovirus 1*	Canine parvovirus Feline panleukopenia virus	CPV-2 FPV	Feces S	Young Young	[260] [241]
	*Carnivore protoparvovirus 2*	Sea otter parvovirus	SoPV	MLN, Li, Lu, S	Unknown	[285]
	*Carnivore protoparvovirus 3*	Canine bufavirus	CBuV	Feces, NP swabs	Unknown	[286]
	*Carnivore protoparvovirus 4*	Fox parvovirus	FoPV	Feces	Unknown	[292]
	*Chiopteran protoparvovirus 1*	Megabat bufavirus 1	BtBuV1	S, feces	Unknown	[355]
	*Eulipotyphala protoparvovirus 1*	Mpulungu (shrew]bufavirus	MpBuV	S, feces	Unknown	[356]
	*Primate protoparvovirus 1*	Bufavirus	BuV	Feces	Unknown	[357]
	*Primate protoparvovirus 2*	Wuharv (rhesus) parvovirus 1	WuBuV1	Feces	Unknown	[358]
	*Primate protoparvovirus 3*	Cutavirus	CutaV	Feces	Unknown	[359]
	*Primate protoparvovirus 4*	Tusavirus	TuV	Feces	Unknown	[360]
	*Rodent protoparvovirus 1*	Minute virus of mice	MVM	Serum	Young	[272]
	*Rodent protoparvovirus 2*	Rat parvovirus 1	RPV1	Tumor	Young	[280]
	*Rodent protoparvovirus 3*	Rat bufavirus SY-2015	RatBuV	I content	Unknown	[334]
	*Ungulate protoparvovirus 1*	Porcine parvovirus	PPV	Fetal tissues	Young	[268]
	*Ungulate protoparvovirus 2*	Porcine bufavirus protoparvovirus [porcine)	PBuV	Feces	Unknown	[294]
*Tetraparvovirus*	*Chiropteran tetraparvovirus 1*	Eidolon helvum (bat) parvovirus 1	BtPARV4	Blood	Unknown	[99]
	*Primate tetraparvovirus 1*	Human parvovirus 4	PARV4	Plasma	Unknown	[300]
	*Ungulate tetraparvovirus 1*	Bovine hokovirus 1	BPARV4	S	Unknown	[308]
	*Ungulate tetraparvovirus 2*	Porcine hokovirus/Porcine parvovirus 3	PPARV4/PPV3	LN, Li, serum, feces	Unknown	[308]
	*Ungulate tetraparvovirus 3*	Porcine parvovirus 2 Parvovirus YX-2010/CHN	PPV2	Serum	Unknown	[301]
	*Ungulate tetraparvovirus 4*	Ovine hokovirus 1	OvPARV4	Li, S	Unknown	[311]
*Chaphamaparvovirus*						
	*Carnivore chaphamaparvovirus 1*	Cachavirus 1 and 2	CachaV-1	Feces	Unknown	[322]
	*Carnivore chaphamaparvovirus 2*	Fechavirus	FChPV	Feces	Unknown	[151]
	*Chiropteran chaphamaparvovirus 1*	Desmodus rotundus chapparvovirus	DrPV-1	K	Unknown	[331]
	*Dasyurid chaphamaparvovirus 1–3*	Tasmanian devil-associated chapparvovirus 1,2, and 6	TdChPV	Feces	Unknown	[332]
	*Galliform chaphamaparvovirus 1*	Turkey parvovirus 2	TPV2	Feces	Unknown	[326]
	*Galliform chaphamaparvovirus 2*	Chicken chapparvovirus 2	ChikPV2	I	Unknown	[327]
	*Galliform chaphamaparvovirus 3*	Chicken chapparvovirus HK				[327]
	*Galliform chaphamaparvovirus 4*	Peafowl parvovirus 1	PePV1	Li, I, heart, stomach	Unknown	[329]
	*Galliform chaphamaparvovirus 5*	Peafowl parvovirus 2	PePV2	Li, I, heart, stomach	Unknown	[329]
	*Primate chaphamaparvovirus 1*	Capuchin kidney parvovirus	CKPV	K	Unkown	[8]
	*Psittacine chaphamaparvovirus 1*	Psittacara leucophthalmus chapparvovirus	PlChPV	Feces	Unknown	[328]
	*Rodent chaphamaparvovirus 1*	Mouse kidney parvovirus Murine chapparvovirus	MKPV MuCPV	K	Unknown	[7] [223]
	*Rodent chaphamaparvovirus 2*	Rat parvovirus 2	RPV2	Feces	Unknown	[334]
	*Ungulate chaphamaparvovirus 1*	Porcine parvovirus 7	PPV7	Feces	Unknown	[315]
	NA	Tilapia parvovirus	TiPV	Feces	Adult	[6]
	NA	Duck associated chapparvovirus	DAC	Oropharyn-geal and cloacal swabs	Unknown	[330]

Abbreviations are those defined by the ICTV or based on the literature. References are of peer-reviewed literature describing either the first detection or first full genome sequence of a virus as recognized by the ICTV

*NA* Not assigned by ICTV up to 2020 [20], *S* Spleen, *Lu* Lung, *K* Kidney, *MLN* Mesenteric lymph node, *Li* Liver, *SI* Small intestine, *I* Intestine, *LN* Lymph node, *CSF* Cerebrospinal fluid, *NP* Nasopharyngeal

**Fig. 2:**
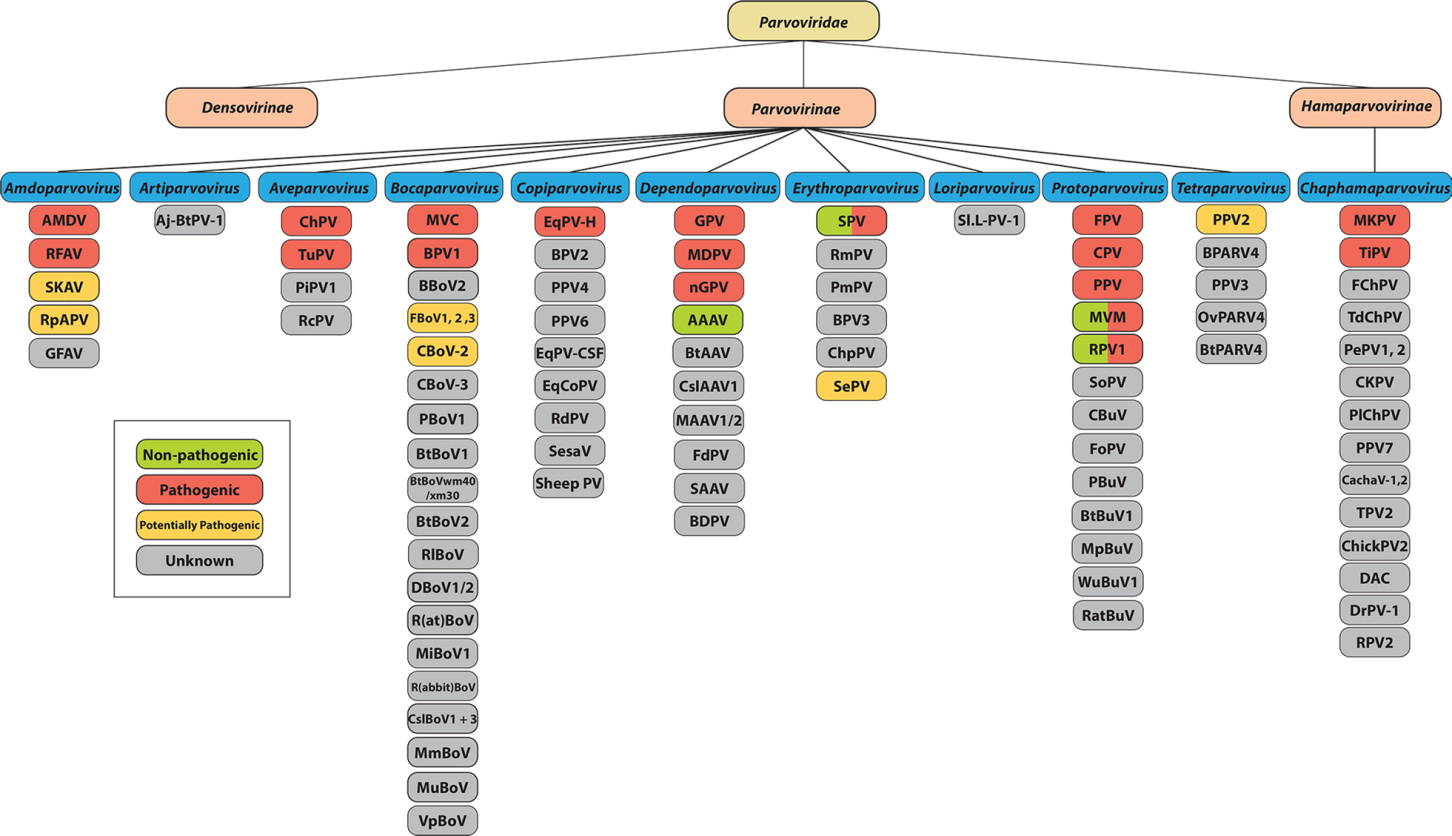
Vertebrate animal Parvovirus Classification and Pathogenicity. A graphical representation of known pathogenicity of select vertebrate animal parvoviruses including all ten genera of the subfamily *Parvovirinae* and one genus of *Hamaparvovirinae*. Viruses marked as both non-pathogenic and pathogenic may have certain conditions, i.e. immunosuppressed host, where the virus is pathogenic. Potentially pathogenic viruses include those where viral nucleic acid has been demonstrated in tissues of a diseased animal or experimental infection has produced disease, but modern Koch’s postulates have not been fully satisfied

**Fig. 3:**
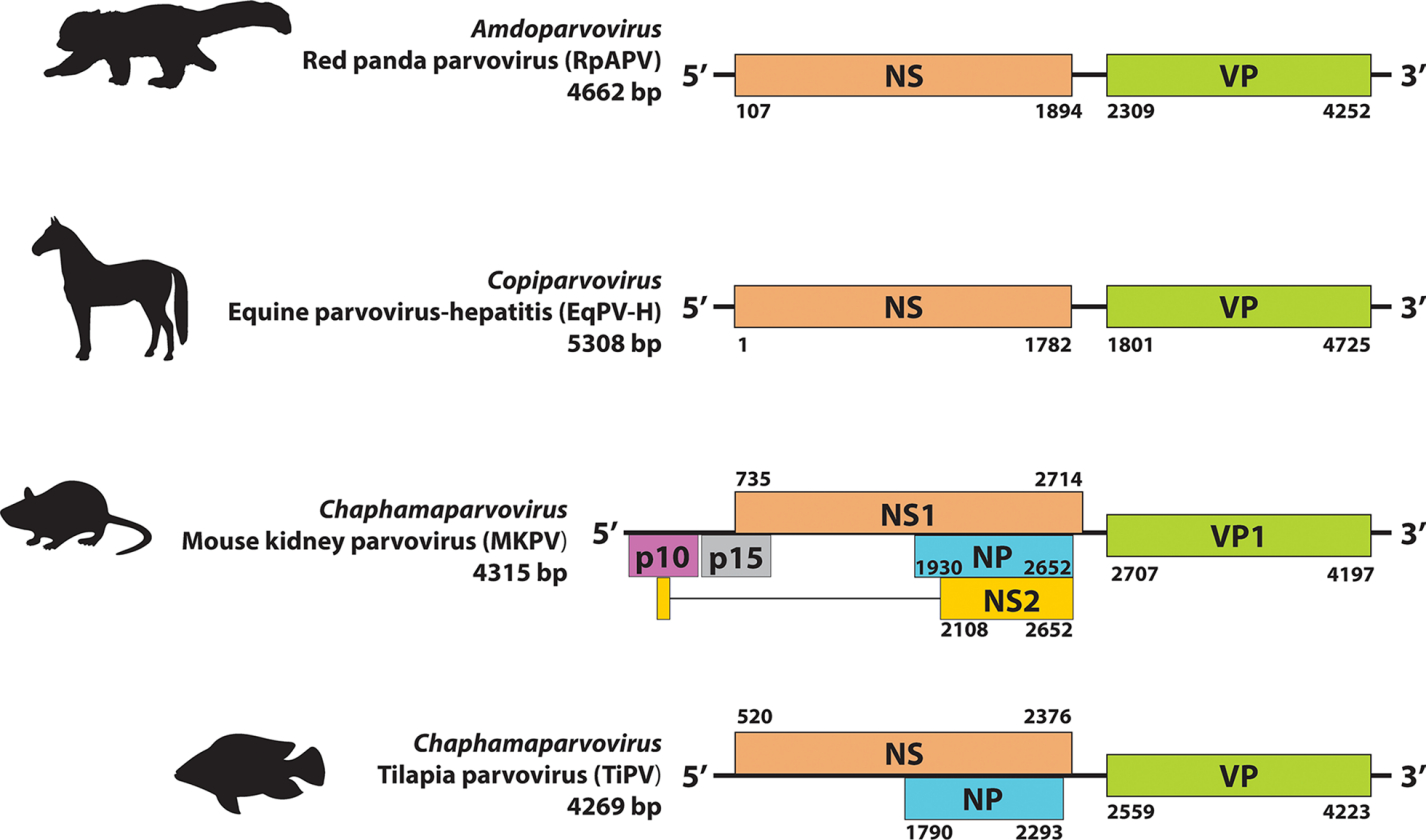
Genome structures of partial or complete coding sequences of recently identified vertebrate parvoviruses. Included are red panda parvovirus (RpAPV) (NC_031751), equine parvovirus-hepatitis (EqPV-H) (MG136722), mouse kidney parvovirus (MKPV) (MH670587), and tilapia parvovirus (TiPV) (MT393593). The genome length known to date, with partial or complete inverted terminal repeats (ITRs), are below the virus name. The colored boxes represent the open reading frames (ORFs) of the non-structural (NS), viral protein (VP), or accessory viral proteins encoded in the genome

Many of these newly discovered viruses are likely part of the complex virome of their host species, and cause little or no disease, while others may be pathogens causing diseases for which an etiological agent has not previously been identified. Also, most parvoviruses likely cause little or no disease in immune competent hosts and the few that are consistently associated with disease appear to be the exception (Fig. 2). Examples of recently identified pathogenic parvoviruses in vertebrate animals include equine parvovirus-hepatitis (EqPV-H), tilapia parvovirus (TiPV), mouse kidney parvovirus (MKPV), and red panda parvovirus (RpPV) [3–10] (Fig. 3). EqPV-H and MKPV represent new parvoviruses associated with long-recognized conditions, whereas TiPV, and possibly RpPV, are of emerging concern.

In situ hybridization (ISH) has been an important tool in identifying disease association of these newly discovered parvoviruses by demonstrating the presence of parvoviral nucleic acids (NA) in lesions [4–7, 10, 11]. This technology, which has been available for decades, has recently been made more accessible through commercialization and rapid probe development. Probes can be designed and produced within weeks after a partial genome sequence is obtained (in contrast to antibody production for immunohistochemistry (IHC) which takes months) [12]. Importantly, ISH allows the use of a modified form of Koch's postulates that was developed in 1996—which states that viral NA sequence should be present within lesions, and thus addresses problems with the classical postulates first developed by Robert Koch and Friedrich Loeffler in 1884, which require isolating, reinfecting, and re-isolating pathogens [13]. As many recently discovered parvoviruses have yet to be isolated from culture, including ISH as a more widely used tool will illuminate the clinical relevance of many of these viruses. Furthermore, experimental infections of free-ranging or endangered species, such as the sea otter or red panda, are not feasible and preclude establishing disease associations for novel viruses through experimental infection studies. Throughout this review, we will present and evaluate the available evidence for pathogenicity of select novel parvoviruses using these guidelines (Table 2).

**Table 2:** Current knowledge on recent parvoviruses of veterinary importance based on Koch’s revisited postulates

	I: genome sequence in most cases of infectious disease	II: fewer/no genome copies in hosts/tissue without disease	III: copy number decreases with disease resolution	IV: sequence detection prior to disease or copy number correlates with severity	V: virus phenotype is consistent with known biological characteristics of its group	VI: in situ hybridization detection of genome copies in tissues with pathology	VII: genome sequence-based evidence is experimentally reproducible	Total number of criteria addressed
Skunk amdoparvo-virus (SKAV)	** + **[72–74, 78, 79]	** + **[72–74, 78, 79]	NA	NA	** + **	** + **[73, 78]	NA	4
Red panda amdoparvo-virus (RpAPV)	NA	NA	NA	NA	** + **	** + **[10]	NA	2
Feline bocaviruses (FBoV1-3)	NA	NA	NA	NA	** + **	** + **[131]	NA	2
Canine bocavirus 2 (CBoV2)	** + **[133–135]	** + **[133–135]	NA	NA	** + **	** + **[135]	NA	4
Equine parvovirus-hepatitis (EqPV-H)	** + **[4, 5, 153, 154]	** + **[4]	** + **[4]	** + **[4]	**−**	** + **[4, 5]	** + **[3, 4]	6
Seal parvovirus (SePV)	NA	NA	NA	NA	**−**	** + **[218]	NA	1
Porcine parvovirus 2 (PPV2)	NA	**−** [281, 283, 285]	NA	NA	** + **	** + **[286]	NA	2
Mouse kidney parvovirus (MKPV)	** + **[7, 8, 11]	** + **[7, 8, 11]	NA	** + **[7, 8, 11]	** + **	** + **[7, 8, 11]	** + **[7, 8, 11]	6
Tilapia parvovirus (TiPV)	** + **[6]	** + **[6]	NA	** + **[6]	** + **	** + **[6]	** + **[6]	6

The seven revisited criteria, adapted from Fredericks and Relman [13], are shown. Evaluation of evidence for pathogenicity was based on total number of criteria addressed for each virus and classified as weak (score 1–2), moderate (score 3–5), or strong (score 6–7)

*NA* not assessed yet/not feasible/not applicable

## General features of parvoviruses

Since the mechanisms of parvovirus replication, genome organization, and structure, have been recently and extensively reviewed elsewhere [14–18], we will only briefly summarize these specific features.

### Taxonomy

The *Parvoviridae* family was originally divided into two subfamilies of viruses that infect either invertebrate (*Densovirinae*) or vertebrate (*Parvovirinae*) hosts, and these subfamilies were then further divided into genera based on their genome organization and amino acid (aa) identity of viral proteins [14]. Viruses are considered members of the same species if their non-structural (NS) proteins share more than 85% aa sequence identity, while diverging greater than 15% from members of other genera [14, 19, 20]. This classification was recently challenged by the discovery of vertebrate parvoviruses, identified via metagenomic sampling of animal feces and named chapparvoviruses, that are more closely related to viruses in the *Densovirinae* subfamily than to those in the *Parvovirinae* subfamily [20]. The International Committee on Taxonomy of Viruses (ICTV) recently split the *Densovirinae* subfamily further into the *Densovirinae* and *Hamaparvovirinae* subfamilies. The latter includes five genera, with the genus *Chaphamaparvovirus* covering these formerly unclassified chapparvoviruses. This new subfamily is characterized by an average of 30% aa sequence identity of their NS1 protein and all species lack the otherwise conserved phospholipase A_2_ domain in the surface viral protein (VP) [20]. Also, parvoviruses can be classified in the same genus if their complete NS1 protein sequence clusters in a monophyletic lineage at the subfamily level, and likewise, for their super family 3 (SF3) helicase domains at the family level [20]. In addition to classification based on genome organization and/or aa sequence identity, parvoviruses can also be classified functionally into either autonomous parvoviruses or dependoparvoviruses, based on their ability to complete their replication cycle independently or their dependence on coinfection with another DNA virus to successfully replicate, respectively.

### Genome organization and major proteins

While differences exist in the presence of accessory proteins, most autonomous parvoviruses are shown as having a genome structure with the large *NS* open reading frame (ORF) on the “left” and the *VP* ORF on the “right” (Fig. 3). The genome termini contain short, imperfect palindromic sequences or inverted terminal repeats (ITRs) that form varying secondary structures, which create self-priming palindromic hairpin telomeres that function as viral DNA replication origins [14]. These secondary structures can either be the same or different at the 5’- and 3’-termini, leading to homotelomeric or heterotelomeric genomes, respectively, and are consistent across a genus. Homotelomeric viruses package equal numbers of plus or minus stranded genomes in viral particles. A packaging bias toward one viral genome strand is observed in parvoviruses with heterotelomeric genomes [21–23].

The *NS* gene (*Rep* in adeno-associated viruses (AAVs) of the genus *Dependoparvovirus*) forms one or more nonstructural proteins (NS1-NS3) via alternative mRNA splicing. The NS1 is a large multidomain protein that plays a central role in DNA replication, as it has strand- and site- specific endonuclease (nicking) activity, an SF3 helicase domain with 3’ to 5’ processivity, rolling circle replication initiator protein motifs, and DNA binding domains. NS1 can also play an essential role in the regulation of the DNA damage response (DDR) and apoptosis pathways, which can be critical for successful completion of the viral infection cycle and contribute to pathology [24].

The *VP* gene (sometimes named *Cap* for the AAVs) encodes the capsid proteins that form the capsid. Parvovirus capsids can be composed of up to 4 VPs (VP1-4) that are generated from a single ORF by alternative splicing and all share a common large C-terminal region. Generally, VP1 is the largest protein and comprises ~ 10% (out of 60 total VP units) of the capsid [14, 15]. In many parvoviruses, the extended N-terminus of the VP1 protein also includes the phospholipase A2 (PLA_2_) enzymatic domain that is essential for cell entry due to the need to release from the endosomal or lysosomal pathway [14, 15]. Additional features of the VP1 N-terminal sequence include a calcium-binding domain (part of the PLA_2_ structure) and a nuclear localization signal [14, 15]. The smallest VP is expressed at a higher rate and comprises the majority of the viral capsid [17]. Capsids range from 22 to 28 nm in diameter with T = 1 icosahedral symmetry, and most have a cylindrical pore at the fivefold axis that is used for genome packaging and exit, as well as for exposure of the N-terminal sequences of the VP2 protein in DNA-containing capsids [14, 15]. These parvovirus capsids can survive for long periods outside the cell, resulting in persistence in the environment, carriage on fomites, and wide dissemination [25, 26].

In addition to the core NS and VP proteins, most parvoviruses express small numbers of ancillary proteins with various functions. For example, a smaller non-structural protein called NP1 is found in the viruses of the genus *Bocaparvovirus* [27]. Other ancillary proteins include NS2 and SAT for minute virus of mice (MVM), 7.5 kDa and 11 kDa for B19 virus, AAP for adeno-associated virus 2, NS2 for Aleutian mink disease virus, and NP in chaphamaparvoviruses [27–32].

### Replication

**Fig. 4:**
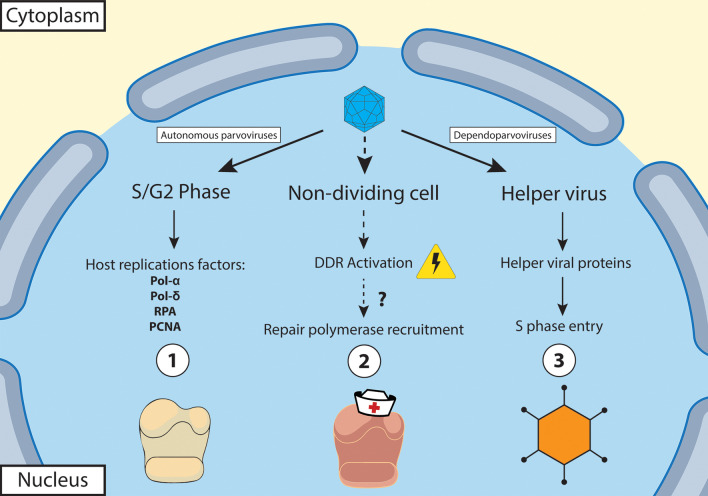
Summary of parvovirus replication requirements. (1) Most autonomous parvoviruses require mitotically active cells (S/G2 phase) to provide host replication factors to replicate their viral genome. (2) Recently, human bocavirus 1 (HBoV1) was demonstrated to replicate in non-dividing airway epithelial cells through hijacking of DNA repair machinery [33, 34]. (3) Dependoparvoviruses depend on co-infection with a helper virus to undergo productive replication in a host cell

Replication of the single-stranded (ss) DNA genome of *Parvoviridae* involves a complex multi-stage process that requires specific host cell conditions that vary by virus and can include cell cycle status, activation of the DNA damage response pathways, or the presence of a helper virus (Fig. 4]. As mentioned above, parvovirus genomes encode few proteins and lack a viral DNA polymerase, and therefore, require host cell enzymes [14, 15]. Unlike some other DNA viruses, like adenoviruses and polyomaviruses, parvoviruses lack the ability to initiate cell division despite their reliance on mitotically active cells with active DNA polymerase and other replication factors to complete their replication [14, 15]. While some dependoparvoviruses are able to complete their infection cycle in both dividing and non-dividing cells due to assistance from their helper viruses, the dependence on the cell cycle status of the infected cell was considered a defining feature of autonomous parvoviruses and plays a significant role in tissue tropism and disease manifestation [14, 15]. However, recent studies have demonstrated that human bocavirus-1 (HBov-1) is able to hijack host cell DNA damage response pathways and the DNA repair machinery to replicate its genome in non-dividing airway epithelial cells (Fig. 4) [33, 34]. Indeed, in all autonomous parvoviruses studied to date, activation of one or more of the DDR PI3-kinase-like kinases is essential for productive infection [14, 35, 36]. Activation of the DDR during infection is also seen in other DNA viruses including papillomaviruses and herpesviruses [37–40].

### Pathogenesis

Many vertebrate parvoviruses have been identified in non-primate species, and the pathogenesis of human parvoviruses such as B19 virus, HBov-1, and human parvovirus 4 have been reviewed elsewhere [41, 42].

While most pathogenic animal parvoviruses affect the young, some, like EqPV-H, appear to cause disease in adults only whereas others, including Aleutian mink disease virus (AMDV), can cause disease in both young and adult animals with different disease manifestations [43, 44]. In general, disease outcome is controlled by various factors. One factor is the need for cellular S phase to access DNA replication machinery and co-factors, so that tissues with a high cellular division rates will be disproportionately affected. Other factors are more complex and include specific regulation of viral transcription and splicing [45–47]. For example, rapidly dividing enterocytes in the epithelial crypts of the intestine of a dog or cat are infected and killed by canine (CPV-2) and feline (FPV) parvoviruses, resulting in enteric disease [48–52], and infection of rapidly dividing cells of the immune system and bone marrow results in panleukopenia in FPV-infected cats. In some instances, the virus is only clinically relevant in immunosuppressed animals, as seen with simian parvovirus infection in macaques [53, 54]. AMDV infection in adult mink, while variable, is characterized by chronic viral replication leading to inflammation and type III hypersensitivity reactions, rather than direct, virally-induced acute necrosis or apoptosis [44, 55–59].

We will now describe some of the parvoviruses of greatest significance in animals, with a special emphasis on emerging and/or novel parvoviruses associated with clinical disease where research progress has been made in the past four years.

## Genus *Amdoparvovirus*

Until 2011, the only known member of the genus *Amdoparvovirus* (formerly *Amdovirus*) was *Carnivore amdoparvovirus* 1, also known as Aleutian mink disease virus (AMDV). Additional species have been identified, including *Carnivore amdoparvoviruses 2–5* (grey fox, racoon dog and fox, skunk, and red panda amdoparvoviruses, respectively), and many others are likely to exist (Fig. 2, Table 1). Recently, a novel amdoparvovirus with 82.7% aa identity to AMDV was identified in rodent pharyngeal and anal swabs[60]. These viruses have a heterotelomeric genome of ~ 4.8 kb with two major ORFs, encoding the nonstructural proteins NS1, NS2, NS3, and the two structural proteins VP1 and VP2 (Fig. 3). The VP1 N termini of amdoparvoviruses are unusually short and lack a PLA_2_ enzymatic domain [61–65].

### Aleutian mink disease virus (AMDV)

Aleutian mink disease (AD), also known as mink plasmacytosis, arose through a combination of economic opportunity and genetic selection. AD was first observed in the US during the 1940s and was initially recognized as a wasting condition in a light-colored mutant of mink called Aleutian mink, based on resemblance with the Aleutian fox coat color that is associated with a lysosomal storage disease (related to Chediak–Higashi syndrome in humans), which likely impairs clearance of immune complexes [66]. Following the commercial success of this new pelt color, Aleutian minks were shipped world-wide. While the wasting condition recognized by the mink farmers was initially attributed to a genetic disease associated with the coat color, subsequent research identified the culprit as AMDV [66, 67].

AMDV causes disease primarily in captive American minks, particularly of the Aleutian coat color, but the host range under natural and experimental conditions includes wild and captive mustelids (weasels, badgers, otters, ferrets), cats, dogs, fox, lynx, racoons, bobcats, skunks, and mice [43, 68–74]. Interestingly, AD has been recognized as an occupational disease of mink farmers [75]. Disease in adult minks is manifested as a persistent infection that leads to immune complex disease characterized by progressive wasting and anorexia, lymphadenomegaly and splenomegaly, proliferative glomerulonephritis, necrotizing arteritis, hypergammaglobulinemia, and plasmacytosis [44, 76]. Anti-capsid antibodies enhance viral entry into macrophages via binding to Fc receptors, resulting in antibody-dependent enhancement of infection [55, 56]. The continuing antibody and viral production leads to the formation of perivascular and glomerular virus-antibody complexes that are deposited in various tissues, leading to type III hypersensitivity with arteritis and glomerulonephritis [55–59]. In young minks, the virus causes an acute disease due to the direct infection and clearance of type II pneumocytes, resulting in fulminant interstitial pneumonia that is often fatal within 3 weeks post-infection [43, 77, 78].

Control was mainly focused on antibody testing and culling, but despite improvements in surveillance testing and biosecurity in recent years, AMDV infections continue globally, likely because of persistence of the virus in the environment [79]. Molecular epidemiology has revealed viral spread within and between farms in multiple countries, and identified regions of increased AMDV prevalence and risky farming practices [80–86]. Recent epidemiologic studies are examining cross-species transmission and viral lineages to identify maintenance hosts that enable viral persistence and viral sources for susceptible species across the globe [87].

### Other amdoparvoviruses

Raccoon dog and fox amdoparvovirus (RFAV) was first recognized following a disease outbreak on six farms raising captive Asiatic racoon dogs and artic foxes in China [88]. Clinical signs in racoon dogs included emaciation, growth retardation, chronic diarrhea, increased thirst, and unkempt fur. Splenomegaly, lymphadenomegaly, and renal cortex congestion were noted at necropsy. Similarly, emaciation and growth retardation were reported in 3-month old artic fox cubs [88].

Skunk amdoparvovirus (SKAV) is related to AMDV, with potential for serological cross-reactivity [89], and early accounts of amdoparvovirus infection in skunks were likely caused by SKAV. Naturally-occurring AD-like disease was first described in two companion striped skunks with biochemical and histologic changes considered typical of AD in mink and ferrets [90]. Several studies have identified the virus or viral DNA in apparently healthy free-ranging striped skunks in both Canada and the US [69, 89, 91–96]. In recent studies, 43/50 (86%) and 140/216 (64.8%) of free-ranging skunks in British Columbia and California, respectively, tested positive for amdoparvovirus DNA[91, 96]. This high prevalence suggests endemic infection in free-ranging skunks. Many PCR-positive skunks had histologic lesions in the kidneys, heart, brain, liver, or lungs similar to the early descriptions of amdoparvovirus in skunks, but only few cases had signs of glomerulonephritis or arteritis [95, 96]. Other clinical signs in both adult and juvenile skunks included neurological signs, emaciation, and lethargy [96]. Although PCR and ISH testing revealed viral NA in liver and spleen of SKAV-infected skunks, providing moderate evidence of pathogenicity [90], elucidating the full tissue distribution of this virus will help confirm its pathogenic role in the variety of observed clinical signs (Table 2).

Red panda amdoparvovirus (RpAPV) was detected by PCR in tissues and feces of six captive red pandas housed at the Sacramento Zoo from 2003 to 2016 [10, 20]. Three animals were healthy when tested, while the other three PCR-positive animals, all geriatric, died and were submitted for necropsy [10]. Histologically, one case presented with inflammatory infiltrates in the mesentery, intestines, pancreas, and myocardium, whereas another case had lytic cells with intranuclear inclusions in oral cavity tissue. ISH and electron microscopy (EM) were performed, and ISH positive cells where present in the germinal centers of lymphoid tissues of all four cases examined, and within scattered individual cells in the lamina propria and mucosa of the intestines of three cases [10]. In one case, hybridization was also consistently detected in areas of inflammation. The morphology of these ISH-positive cells was suggestive of macrophages. EM of lingual epithelial tissue demonstrated capsids of ~ 22 nm in diameter, consistent with other parvoviruses [14, 15]. These findings provide weak evidence of pathogenicity, and additional studies examining the prevalence, diversity, and pathogenicity, of RpAPV in captive and free-ranging red pandas are needed to clarify the significance of this virus for global conservation efforts (Table 2). Red pandas are listed as endangered by the International Union for Conservation of Nature and considered threatened with extinction by the Convention on International Trade in Endangered Species, so experimental studies of RpAPV infection and disease are not possible [97].

Gray fox amdoparvovirus (GFAV) was first detected in a gray fox in California in 2009 [98]. This animal presented with severe gait abnormalities, lymphadenopathy, and acute muscle inflammation, and was subsequently euthanized. GFAV was also detected in the lung and heart tissues of an additional fox with similar signs. However, nine other foxes with similar clinical signs tested negative for GFAV in this study, and thus, it is likely that the DNA was an incidental finding in the two foxes that tested PCR positive.

Recently, two new amdoparvoviruses were detected in various tissues of Canadian carnivores [74]. Labrador amdoparvovirus 1 (LaAV-1) was identified in foxes and martens and Labrador amdoparvovirus 2 (LaAV-2) was identified in one fox. LaAV-1 was most similar to viruses of mink and skunks, with capsid proteins in some regions almost indistinguishable from those of AMDV [74]. In contrast, LaAV-2 was more closely related to other viruses infecting canids.

## Genus *Artiparvovirus*

This new established genus currently includes only one member, Artibeus jamaicensis parvovirus (Aj-BtPV-1, *Chiropteran artiparvovirus 1*) (Fig. 2) [99]. This virus was detected in whole EDTA blood samples of leaf-nosed fruit bats in Panama and is currently not associated with clinical disease.

## Genus *Aveparvovirus*

The genus *Aveparvovirus* is composed of three official members, all of which have been detected in avian species (Fig. 2, Table 1). The species *Galliform aveparvovirus* 1 includes two viral strains, chicken parvovirus (ChPV) and turkey parvovirus (TuPV), which are widespread and highly infectious to young poultry, although their disease association remains uncertain. The other species are *Columbid aveparvovirus 1* (pigeon parvovirus 1) and *Gruiform aveparvovirus 1* (red-crowned crane parvovirus), in addition to a recently identified aveparvovirus in a grey pileated finch. Aveparvoviruses lack a PLA_2_ motif in their VP1 proteins. Since avian parvoviruses have been recently reviewed elsewhere [100], we will only describe their main features here, emphasizing recent findings.

### Chicken (ChPV) and Turkey (TuPV) parvoviruses

These two viruses are widespread amongst commercial poultry, including the US, Hungary, Poland, and Croatia [101–105], and ChPV has also been reported in South Korea, China, India, Brazil, Ecuador, Canada, and the UK [106–112]. While ChPV and TuPV have been associated with malabsorption syndrome in chickens and the occurrence of enteritis in turkeys, respectively, the contribution of these viruses to disease remains unclear as they are also detected in healthy birds [103, 105, 113–119]. Runting and stunting syndrome (RSS) in broilers is a multifactorial condition first reported in the 1940s, characterized by impaired growth and poor feed conversion because of enteritis, and is associated with several other viruses [e.g. astrovirus, coronavirus, rotavirus, reovirus), bacteria, and coccidia [111, 113, 120–124].

Experimental infection of specific-pathogen-free 1-day old chicks with ChPV resulted in rapid development of enteritis and diarrhea that persisted until 42 days of age, whereas mock-inoculated birds did not develop any clinical signs or macroscopic lesions [123]. Infected birds in all age groups presented with intestinal volvulus, a feature reported in RSS cases, and developed enteritis characterized by dilated crypts and acute pancreatitis [123]. Although many questions remain about the relationship between ChPV and RSS, a causal relationship between ChPV and enteric disease in young chickens seems likely.

### Other aveparvoviruses

Analysis of aveparvovirus DNA in the droppings of wild pigeons in Hong Kong and Hungary showed a close, but distinct, relationship with ChPV and TuPV and was named pigeon parvovirus 1 (PiPV1) [125]. A study in Brazil identified another parvovirus sequence in the droppings of a gray pileated finch, tentatively named Passeriform aveparvovirus [126]. Red-crowned crane parvovirus (RcPV) was detected in a fecal virome of wild and captive red-crowned cranes in China [20, 127]. These three aveparvovirues were detected by sequencing and PCR, and any potential disease association is unknown.

## Genus *Bocaparvovirus*

The genus *Bocaparvovirus* (formerly *Bocavirus*) is the largest in the *Parvoviridae* family and currently includes over 25 species from many animal hosts, such as carnivores, bats, and ungulates (Fig. 2, Table 1). Human bocavirus 1 (*Primate bocaparvovirus 1, HBoV-1*) was first detected in 2005 in the nasopharyngeal aspirates of children with respiratory tract infections, and is associated with respiratory tract infections and acute otitis media in young children [41, 128]. Recent discoveries in this genus include two new *Ungulate bocaparvovirus* species (*Ungulate bocaparvovirus 7 and 8*) and two new *Rodent bocaparvovirus* species (*Rodent bocaparvovirus 1 and 2*) [20]. Bocaviruses have heterotelomeric genomes ~ 5 to 5.5 kb and package predominantly negative-sense DNA [27, 129, 130]. Bocaparvoviruses encode for an NP1 protein that plays a role in RNA processing, as mentioned previously [131, 132]. Most bocaparvoviruses were discovered after 2010 by metagenomic analysis of fecal DNA (Table 1). While many of these new viruses are widespread, none have been unequivocally demonstrated to be pathogenic in animals (Fig. 2).

### Minute virus of canines (MVC)

MVC (*Carnivore bocaparvovirus 1*), also known as Canine minute virus (CnMV) or canine parvovirus type-1 (CPV-1), was first isolated from feces of healthy dogs in 1967 and has since been associated with a range of pathologies in dogs of different ages [133]. Initial in vivo experimental inoculations suggested that MVC was apathogenic, but a virus with fewer in vitro passages caused severe respiratory disease in 5-day old puppies [134]. Natural outbreaks of MCV-associated neonatal mortality have also been reported [134–136]. Gross lesions in these naturally infected puppies included diarrhea and pale streaks in the heart. Histologic findings of large eosinophilic intranuclear inclusions in jejunal enterocytes, intestinal crypt necrosis, lymphoid depletion, pneumonia, and myocardial necrosis, were also described [135]. Transplacental transmission in pregnant dogs has been described and can result in embryonic resorption, abortion, stillbirth, birth deformities, or neonatal mortality [137–139].

### Bovine bocaparvoviruses (BPV)

The hemadsorbing enteric virus BPV1 (*Ungulate bocaparvovirus 1)* has been recognized for decades and was first discovered in 1961 in the gastrointestinal tract of calves with diarrhea [140], but appears to be widespread in both healthy and diarrheic calves. In calves, BPV1 can cause watery to mucoid diarrhea following infection of enterocytes throughout the small intestine. It has also been associated with spontaneous abortions and stillbirths in adult cattle [141]. Like some other pathogenic parvoviruses, BPV infection will start with initial replication in the tonsils and gut and spread to lymphoid organs where it will result in transient lymphopenia. Viral antigen has been detected in multiple tissues including the epithelium of intestinal crypts, thymus, lymph nodes, adrenal glands, and heart [142]. BPV1 has been analyzed with high resolution X-ray and cryo-EM, providing a model for other members of this genus [15].

More recently, the DNA of BPV2 (*Ungulate bocaparvovirus 6)*, which shares 64% NS1 aa identity with BPV1, was detected in nasal swabs of cattle in the US and Mexico in 2015 [143]. However, the prevalence was equal in cattle that were either healthy or suffered from acute bovine respiratory disease, so disease association is currently unknown.

### Feline bocaviruses (FBoV)

DNA of FBoV (currently FBoV1 to FBoV3) was recently discovered in domestic cats and these viruses have been assigned to *Carnivore bocaparvovirus *3–5, respectively. FBoV1 was first detected in feces, blood, kidney, and nasal swabs of clinically normal domestic cats in Hong Kong in 2012 [144]. FBoV2 and FBoV3 were subsequently discovered in a high throughput metagenomic study of feline fecal viromes [145, 146]. FBoV have been detected in the feces of symptomatic and asymptomatic cats in Portugal, Japan, China, Belgium, Thailand, and the US [145–150]. Testing for FBoV in feces of stray cats with and without signs of diarrhea in China yielded one positive case, a three-month-old male cat with severe enteritis [129]. FBoV1 was detected in cats from different households with hemorrhagic enteritis during an outbreak of feline panleukopenia virus (FPV) in Thailand [150]. Using ISH, parvoviral NA was detected in intestinal cells and vascular endothelium of the intestinal mucosa and serosa, with co-infecting FPV detected via IHC. The coding sequences revealed high inter-strain genetic diversity and possible NS1 recombination in one of three FBoV1 strains [150]. One year later, FBoV1 to -3 were detected in the feces of cats during an outbreaking of vomiting and diarrhea in a system of shelters in British Columbia, Canada [151]. Although the pathogenicity of FBoV in cats remains unclear based on weak evidence, the presence of parvoviral NA in the intestine of cats with hemorrhagic enteritis [150] suggests a pathogenic association of this virus, alone or in combination with other viruses such as FPV (Table 2). The common finding of the virus or viral DNA in healthy cats suggests mostly subclinical infections, with disease under some circumstances.

### Other bocaparvoviruses

The DNA of two novel bocaviruses has been reported in dogs in recent years, although without any definitive association with clinical disease. Canine bocavirus 2 (CBoV2) of the species *Carnivore bocaparvovirus 2* was discovered during a metagenomic study of dogs with respiratory disease and has also been detected in fecal samples from stray dogs in a surveillance program and in a litter of puppies with fatal enteritis [144, 152, 153]. Although CBoV2 has been detected more frequently in dogs with respiratory disease compared to healthy dogs, a clear connection with pathology has yet to be established. A novel strain of CBoV2 was identified in a litter of puppies that died of acute dyspnea and hemoptysis using next generation sequencing (NSG) and the tissue distribution was assessed using qPCR and ISH [154]. Hybridization was detected in intestinal epithelial cells and EM confirmed the presence of particles within intranuclear inclusions of small intestinal enterocytes. Nonetheless, it remains unclear what role this infection played in the death of the puppies and the evidence of pathogenicity is weak (Table 2). Canine bocavirus 3 (CBoV3) was discovered in the liver of a dog co-infected with a novel circovirus [155].

Porcine bocavirus 1 (PBoV1) was first discovered in the lymph nodes of pigs affected by post-weaning multisystemic wasting syndrome (PMWS) in Sweden in 2009 [156], and has been detected worldwide in both healthy and clinically ill pigs. Its epidemiology, evolution analysis, detection methods, and pathogenesis have been recently reviewed elsewhere [157]. While the pathogenicity of PBoV is unclear, it has been detected in association with a wide array of clinical signs, and co-detected with other viruses such as porcine circovirus type 2, porcine reproductive and respiratory syndrome virus, and torque teno sus virus [158–160].

In the last decade, several new species of bat bocaviruses have been discovered through metagenomic analyses of various bat species. Myotis myotis bocavirus 1 (BtBoV1, *Chiopteran bocaparvovirus 1*) was detected in pharyngeal and anal swabs from 11 insectivorous bat species in China in 2012 [161]. The bat bocaviruses WM40 and XM30 (*Chiopteran bocaparvovirus 2 and 3*, respectively), were detected in organs of insectivorous bats in Myanmar in 2013 [162]. Miniopterus schreibersii bat bocavirus (BTBoV2) of the genus *Chiopteran bocaparvovirus 4* was discovered in spleen, respiratory, and alimentary, samples of six bat species in China in 2017 [163]. A higher prevalence of BTBoV2 in the bat species *Rhinolophus sinicus* led the authors to suggest that this bat could be the primary viral reservoir. BTBoV2 was also more frequently detected in female bats and prevalence was higher during lactating seasons. The same authors performed a similar study one year later and identified Rousettus leschenaultii bocaparvovirus 1 (RIBoV, *Chiopteran bocaparvovirus 5)*, as well as a few other novel bat bocaviral sequences [164].

Other Bocaparvovirus DNA has been found in dromedary camels, rats, mink, rabbits, sealions, as well as other animal species (Table 1). The dromedary camel bocaparvoviruses (DBoV)1 and -2 (*Ungulate bocaparvovirus 7* and *8*, respectively), were discovered through a metagenomic analysis of dromedary camels in Dubai [165]. Interestingly, while *NS1*/*NP1*/*VP1-2* genes were observed in both viruses, a phospholipase A_2_ motif was not detected in the VP1 sequences of 18 isolates and no start codons were found for the VP1 ORF [165]. Rat bocavirus (RBov, *Rodent bocaparvovirus 1*) DNA has been detected in the alimentary and respiratory tracts, spleen, and kidneys, of Norwegian brown rats in China [164, 166]. Mink bocavirus 1 (MiBoV1, *Carnivore bocaparvovirus 6*) was detected in feces of healthy and sick mink from a breeding center [167]. Rabbit bocaparvovirus (RBoV, *Lagomorph bocaparvovirus 1*) DNA was discovered in the feces of rabbits with enteric disease, but association with disease is not clear [168]. California sea lion bocavirus (CslBoV)1 and -3 (*Pinniped bocaparvovirus 1 and 2*, respectively) DNA has been detected among fecal samples collected from free-ranging California sea lions in California [169].

## Genus *Copiparvovirus*

Viruses in this genus have only been identified in mammals thus far, the clinical significance of most remains in question, and none have been cultured in vitro (Fig. 2, Table 1). Copiparvoviruses were first identified in domestic cows and pigs using DNA sequencing, then equine parvovirus-hepatitis (EqPV-H) was identified in horses. The genomic organization includes *NS* and *VP* genes, and genome lengths vary between 5.3 and 5.9 kb [170] (Fig. 3). Detailed information on protein expression and/or telomere structure are not yet available.

### Equine parvovirus-hepatitis (EqPV-H)

Fulminant acute hepatic necrosis in horses was first described in 1918 by Sir Arnold Theiler and became known as Theiler’s disease [171]. Thousands of horses were vaccinated for African Horse Sickness by administration of pooled convalescent horse serum along with live virus. Four to 24 weeks after treatment, 2–18% of the horses succumbed to hepatic necrosis. Some unvaccinated horses living with vaccinated horses also developed hepatic necrosis, while horses on properties without vaccination had less or no hepatitis. These findings suggested the condition to be both transmissible and contagious, although an infectious agent was not identified.

One hundred years later, in 2018, EqPV-H was identified by NGS of a liver sample from a horse that died of Theiler’s disease [3], and was confirmed to be the primary pathogen in Theiler’s disease cases through prospective case series [172, 173]. Examination of the tissue distribution of viral DNA by qPCR and ISH, demonstrated that the virus is hepatocytotropic [4]. It appears that EqPV-H mostly causes subclinical or mild hepatitis, with the fulminant hepatic necrosis (Theiler’s disease) being a rare outcome [4]. Pathologic findings in mild cases include individual hepatocyte necrosis and lymphocytic infiltrates [4], while severe cases have centrilobular-to-massive hepatocyte necrosis with variable inflammatory infiltrate, vacuolar changes in spared portal areas, and biliary reaction [172, 173]. A prolonged period of high viremia before the onset of hepatitis suggests that the virus might not be directly cytolytic, but it remains to be determined whether pathology is a direct effect of the virus or is immune-mediated.

EqPV-H is present worldwide and both viral infection and presence of viral DNA are common in horses, with a serum PCR prevalence of 8–37% and a seroprevalence of 15–35% [3, 5, 174–179]. qPCR analysis of historical samples of horses with Theiler’s disease from 1981 demonstrates that this virus has been circulating at least 40 years (Dr. Thomas J. Divers, personal communication). Transmission via iatrogenic administration of equine biologic products is well documented, and other modes of transmission are being explored [4]. A seasonal pattern of non-iatrogenic Theiler’s disease cases in the summer and fall suggests an insect vector [172, 173], although a horse fly transmission study did not result in viral spread [4]. Oral, nasal, and fecal shedding of viral DNA has been demonstrated and successful oral transmission of one single horse reported, however, the primary route of transmission is not yet known. Vertical transmission has not been observed [4]. While these findings provide strong evidence of pathogenicity, much remains to be determined, including what governs tissue tropism and influences disease severity (Table 2).

### Other copiparvoviruses

The genus *Copiparvovirus* includes bovine parvovirus 2 (BPV2) and porcine parvovirus 4 (PPV4) (*Ungulate copiparvovirus 1* and *2*, respectively) [170, 180]. BPV2 DNA was found in bovine sera, including pooled serum samples of calves in the US [180, 181]. BPV2 DNA was also detected in metagenomic analysis of bovine pneumonic lung samples in Canada, although its presence was not significantly associated with pneumonia [182]. Other bovine copiparvoviruses have been identified in bovine and fetal bovine serum, including the bovine copiparvovirus species 3 isolate JB9 [183] and Bosavirus (BosaV, *Ungulate copiparvovirus 5*) [181], respectively. These findings suggest bovine parvovirus DNA, and perhaps virus, might be present in many products produced using bovine serum.

PPV4 DNA was initially detected in lung lavages of pigs infected with porcine circovirus type 2 in the US and has subsequently been detected in a variety of tissues of both healthy and sick pigs in the US, China, Vietnam, Thailand, Japan, Poland, Hungary, South Africa, and Cameroon [184–191]. PPV4 has also been detected in bush pigs in Uganda and wild boar in Romania and South Korea [187, 192, 193]. The pathogenicity of PPV4 remains unclear. Porcine parvovirus 6 (PPV6, *Ungulate copiparvovirus 4*) was first identified in aborted pig fetuses in China, with a higher prevalence in aborted pig fetuses and piglets compared to finishing pigs and sows [194]. Later, PPV6 was detected in serum samples from pigs in nine US states and one state in Mexico [195]. However, the clinical significance of the virus remains undetermined.

In horses, two other copiparvoviruses have been identified to date. The first was discovered in a cerebrospinal fluid (CSF) sample from a horse with neurological signs and leukocytic pleocytosis in 2015 [196]. This viral DNA, named horse parvovirus-CSF, was detected in thoroughbreds in China in 2018 and in metagenomics analysis of samples from horses with unexplained neurological or respiratory signs [178, 197]. In the latter study, another copiparvovirus was identified, tentatively named equine copiparvovirus (EqCoPV) and its NS1 protein shares 43.4% and 31.3% aa identity to horse parvovirus-CSF and EqPV-H NS1, respectively [178].

Roe deer copiparvovirus (RdPV*, Ungulate copiparvovirus 3*) DNA was identified through metagenomic sequencing of *Ixodes ricinus* ticks and European roe deer in Belgium [198]. The RdPV genome encodes for two putative ORFs and its *NS* gene has 55% nucleotide identity to BPV2, its closest relative. Importantly, the presence of the virus in both deer and ticks suggests a role for ticks in transmission of these parvoviruses, although that has not been confirmed.

Sesavirus (SesaV, *Pinniped copiparvovirus 1*) is a non-ungulate copiparvovirus and its DNA was detected in the feces of a California sea lion pup with malnutrition and pneumonia [199]. The identity of the putative SesaV NS and VP proteins to the closest members of the genus are only 25% and 28% aa, respectively. The putative sheep copiparvovirus 1 (Sheep PV) was detected in metagenomic sequences of samples from an abortion outbreak in sheep, and shares < 30% identity with the NS aa sequence of other copiparvoviruses and < 20% identity with members of other genera in the *Parvovirinae* family [200].

## Genus *Dependoparvovirus*

Viruses in this genus, formerly known as *Dependovirus*, are phylogenetically most similar to the genera *Copiparvovirus*, *Erythroparvovirus*, *Artiparvovirus, Loriparvovirus,* and *Tetraparvovirus*. Most of its members are adeno-associated viruses (AAVs), which require helper viruses to complete their viral infection cycle (Fig. 2, Table 1) [100]. However, the goose parvovirus (GPV) and Muscovy duck parvovirus (MDPV) (*Anseriform dependoparvovirus 1*), are autonomous and replicate in tissues of growing goslings or ducklings [100]. Differences are seen in the organization of the genomes, with AAVs typically having identical, short terminal repeats of ~ 145 bp long with T-shaped hairpin telomeres of ~ 125 bp, whereas GPV and MDPV have long identical telomeres of 442 to 456 bp, respectively [201].

The prototypical dependoparvovirus is the human adeno-associated virus 2 (AAV2), first discovered in the mid 1960s in contanimated laboratory adenovirus preparations [202, 203]. In the section “Animal viruses as therapeutics in humans”, we will discuss the use of veterinary parvoviruses to replace human AAVs in efforts to improve gene therapy performance.

### Goose parvovirus (GPV) and Muscovy duck parvovirus (MDPV)

GPV was first identified in China in 1962 and later in Hungary in 1967 [204, 205]. Disease is most severe in goslings and can result in a high mortality of > 90% in birds less than 4 weeks old. Gross lesions can include fibrinous pseudomembranes of the oral cavity, pericarditis, pulmonary edema, and catarrhal enteritis, and microscopic lesions include intranuclear inclusions and degenerative changes in myocardial cells [206]. MDPV is closely related to GPV with 87% nucleotide identity with GPV at the genome level and 91.2% aa homology of the Rep (NS) protein [207] MDPV causes Muscovy duck parvoviral disease, first described in China in 1984 and characterized by respiratory signs, diarrhea, and dyskinesis, with a lower morbidity and mortality rate compared to GPV. This disease most commonly occurs in 3-week-old Muscovy ducklings and is commonly known as the “3-week” disease [201]. In more recent years, a recombinant MDPV (rMDPV) was identified in Chinese Muscovy ducks with a higher mortality and a catarrhal disease outcome similar to GPV [208, 209].

In 2015, a novel goose parvovirus (nGPV) was described in China in association with growth retardation, beak atrophy, enteritis, and paralysis [210–212]. Phylogenetic analysis revealed that these Chinese nGPV strains share 90.8–94.6% nucleotide identity with GPV and thus, are closely related [210]. While the disease, named short beak and dwarfism syndrome (SBDS), has a reportedly low mortality, the morbidity can be up to 20%. More recently, SBDS has also been observed in Egypt and Poland [213, 214].

### Other dependoparvoviruses

Many other AAVs have been discovered, and an avian AAV (AAAV) was first discovered in 1973 and subsequently cloned into recombinant vectors for use in avian gene therapy studies in postmitotic avian cells [215, 216]. These recombinant AAVs can be used to transduce avian neurons and retinal cells for manipulation of gene expression [217]. AAAV was used for the development of a vaccine against duck hepatitis A virus-1 [218].

Bat adeno-associated virus (BtAAV, *Chiropteran dependoparvovirus 1*) was identified in fecal swabs of 19 bat species in five Chinese provinces in 2007–2008 [219]. Intestinal samples from 5 species of bats in Southeast China showed that 18.6% were positive for AAVs, suggesting a wide distribution of these viruses [220]. Analysis of the BtAAV capsid structure using sequence analysis and cryo-EM revealed unique structural differences to human AAVs, including insertions and deletions in the capsid surface loops [221]. BtAAV capsids have been identified as possible vectors for gene therapy, as discussed later [221, 222].

Other dependoparvoviral DNA detected in fecal samples include (1) California sea lion adeno-associated virus 1 (CslAAV1, *Pinniped dependoparvovirus 1*), in feces of sea lions in California [169], (2) Murine adeno-associated virus (MAAV)1 and -2 (*Rodent dependoparvovirus 1 and 2*), respectively, in feces of house mice in New York City [223], and (3) Feline dependoparvovirus (FdPV, *Carnivore dependoparvovirus 1*), detected in two cats in an outbreak of vomiting and diarrhea in a shelter in British Columbia, Canada [151].

This genus also includes the only known reptilian parvoviruses, such as snake AAV (SAAV, *Squamate dependoparvovirus 1*), which has been isolated from a ball python, propagated in viper and iguana heart cells, and fully sequenced [224]. Partial genome sequences of a dependoparvovirus were detected in an Indonesian pit viper [225] and a chequerboard worm lizard [226]. A dependoparvovirus was detected in a bearded dragon (BDPV, *Squamate dependoparvovirus 2*) [227]*.* No diseases have been associated with any of these reptilian parvoviruses.

## Genus *Erythroparvovirus*

The genus *Erythroparvovirus* (formerly *Erythrovirus*) includes viruses detected in a wide range of species including primates, seals, cattle, and rodents (Fig. 2, Table 1). Human parvovirus B19 (B19V) infection is common in humans, and can be either asymptomatic or symptomatic, with a wide range of clinical diseases including erythema infectiosum (fifth disease) in children, chronic arthropathy in adults, hydrops fetalis, and transient aplastic crisis [228–230]. Recently, a B19V-related virus was detected in free-ranging and captive new world primates in Central America [231]. Simian parvovirus (SPV, *Primate erythroparvovirus 2*) shares features with B19V, including its molecular structure and tropism for the bone marrow, but most infections are non-pathogenic except in immunosuppressed or anemic monkeys [53, 54]. SPV was discovered in 1992 when immunosuppressed macaques developed severe anemia. Histology revealed decreased erythroid and myeloid lineages with occasional intranuclear viral inclusions, and EM showed intranuclear viral particles characteristic of parvoviruses [53]. In 2000, erythroparvoviruses were identified in the serum of captive nonhuman primates with anemia, including rhesus macaque parvovirus (RmPV, *Primate erythroparvovirus 3*) and pig-tailed macaque parvovirus (PmPV, *Primate erythroparvovirus 4*) [232].

Bovine parvovirus 3 (BPV3, *Ungulate erythroparvovirus 1*) was first detected in bovine serum [180], and later in cattle in Brazil [126], but no disease has been associated. Chipmunk parvovirus (ChpPV, *Rodent erythroparvovirus 1*) was identified in the sera of Manchurian chipmunks in Korea [233], and its NS1 induces apoptosis in COS-7 cells, similar to what has been observed with NS1 of other parvoviruses [234]. No disease has been associated with ChpPV infection.

Seal parvovirus (SePV, *Pinniped erythroparvovirus 1*) DNA was detected in brain tissue of a harbor seal with non-suppurative meningoencephalitis in the Netherlands [235]. ISH revealed viral NA in the cerebral parenchyma adjacent to the meninges, suggesting a potential association between the virus and lesions. Analysis of archived tissues (1988–2014) from harbor and grey seals demonstrated that SePV DNA was present in both populations [236]. Currently, these findings provide weak evidence to support pathogenicity and are limited by the feasibility of experimental infections of marine mammals (Table 2).

## Genus *Loriparvovirus*

Slow loris parvovirus 1 (SI.L-PV-1, *Primate loriparvovirus 1*) DNA was detected in the serum and organs of a captive 22-year-old slow loris, a small nocturnal primate, that was euthanized due to poor condition following diagnosis with histiocytic sarcoma [237]. The virus was detected in banked serum samples of that animal collected over an 8-year period prior to death, demonstrating persistent infection, but samples from 25 other animals yielded negative results [237].

## Genus *Protoparvovirus*

Members of the genus *Protoparvovirus* (formerly *Parvovirus*) package a genome of ~ 4.5–5.5 kbp and encode two large genes that encode for NS1 and VP1/2 [14]. These viruses infect a wide range of animal hosts and cause a variety of conditions from subclinical to lethal disease (Fig. 2, Table 1) [14]. Viruses include cutavirus (CuV), infecting humans; porcine parvovirus (PPV), infecting pigs; minute virus of mice (MVM), infecting rodents; feline panleukopenia virus (FPV) and canine parvovirus 2 (CPV-2) which infect members of the Order Carnivora, including cats, racoons and mink. CPV-2 infection of rescued, free-ranging Taiwanese pangolins, provides the first evidence of CPV-2 infection in a non-carnivore [238, 239]. CPV-2 arose as a variant of FPV, creating pandemics among dogs, coyotes, and wolves [19], and we will discuss both viruses as a model for understanding emergence and host-switching.

### Feline panleukopenia virus (FPV)

Publications from the 1920-30 s reported an infectious enteritis in cats and raccoons with a high mortality rate referred to by various names including malignant panleukopenia, infectious agranulocytosis, and more recently, feline panleukopenia [240]. The infectious agent for this disease, named FPV, was first isolated in tissue culture from a captive snow leopard in the 1960s after it was recognized it required dividing cells for replication [241]. Shortly thereafter, additional viruses were isolated from cats and other hosts, and some of those early isolates were used to prepare attenuated viral strains by passaging in tissue culture [242]. Those attenuated viruses were soon included in the standard vaccines that are now recommended for all kittens [243]. Due to the vaccination program, FPV became a less frequent disease in many countries including the UK, Australia, New Zealand, and the US. Diseases caused by FPV include enteritis in kittens older than ~ 6 weeks (as younger animals are generally protected by maternal immunity), often accompanied by panleukopenia, due to virus replicating in the rapidly dividing cells of the intestinal crypts, bone marrow, and other lymphoid tissues [48, 49]. Another disease outcome is seen when neonatal kittens are infected, where virus infecting the cerebellum causes cerebellar hypoplasia and ataxia [244]. Recent work has revealed the structural features of the FPV capsid [245], the host-antibody responses [246], and the genetic variation in FPV sequences from many parts of the world [87, 233, 247–251]. Those studies have revealed the overall evolution of FPV, and helped to explain processes that underlie viral emergence, viral adaptation, and genetic variation.

### Canine parvovirus (CPV-2)

Canine parvovirus emerged as a new pathogen in dogs during late 1970s as the result of a cross-species transmission event from an FPV-like virus. The successful cross-species transfer and adaptation to the new canine host involved around six aa changes on the surface of each capsid copy/subunit, which allowed the virus to bind and infect cells using the canine cellular transferrin receptor type-1 [52, 252, 253]. The original strain that emerged in 1978, CPV-2, was replaced one year later by a variant termed CPV-2a, which contained 5 additional mutations in the capsid protein, regaining the feline host range and also likely allowing CPV-2a to infect other hosts including minks, coyotes, foxes, and raccoons. Although CPV-2 and FPV are over 98% identical in DNA sequence, they differ in host range, antigenic structure, and hemagglutination properties [252, 254]. CPV-2 targets rapidly dividing cells in puppies, including those in intestinal epithelial crypts, bone marrow, lingual epithelium, oral cavity, and cardiac myocytes [50]. After an incubation period of 3–7 days, clinical signs may include vomiting, hemorrhagic diarrhea, depression, lymphopenia, loss of appetite, fever, and dehydration in younger dogs. Infection in neonatal puppies, can result in myocarditis after a few weeks [48, 51, 255–260]. CPV-2 can be fatal when untreated, but infection is prevented by vaccination [260–263]. The emergence of CPV-2 as a pandemic virus in the late 1970s represents one of the few accessible models of a virus jumping to a new host where we can compare in detail the ancestral and descendent viruses to better understand fundamental processes that control host selection, emergence, and viral evolution. After 40 years of viral spread, new sequencing and imaging approaches have revealed natural variants and the capsid features that bind antibodies and the host receptor, leading to viral adaptation and host switching [264–267].

### Porcine parvovirus (PPV)

PPV (*Ungulate protoparvovirus 1*) causes a series of conditions in fetal pigs termed “SMEDI” (stillbirth, mummification, embryonic death, and infertility). PPV is endemic in most countries, and severe outbreaks can occur in unvaccinated herds that lack maternal immunity. PPV was first detected in the early 1960s in primary porcine kidney and testicle cell cultures used to cultivate hog cholera virus [268]. The virus was only later detected in a series of stillbirths, infertility, and abortions in a pig herd [269]. For more information on PPV, we refer to excellent recent reviews [270, 271].

### Minute virus of mice (MVM)

MVM (*Rodent protoparvovirus 1*) has been used to better understand parvovirus biology and replication. First discovered in 1966, MVM naturally infects laboratory and wild mice, and hamsters and rats can be experimentally infected [272–274]. Infections are mostly subclinical and clinical disease in mice is generally limited to experimental infections with certain strains of virus. MVM infections can alter T lymphocyte functions [272, 275–277]. Two variant MVM strains were isolated from contaminated cell cultures and named MVMp (for prototype) and MVMi (for immunosuppressive). Experimental infection of adult BALB/c and C57BL/6 mice with either strain is asymptomatic, but MVMi causes growth retardation and failure to develop effective circulating antibody titers against the virus in neonatal mice [278]. The MVM NS1 protein mediates the localization of the viral genome to sites of DNA damage during viral replication [279], and there is a close relationship between viral replication and DNA damage pathways that likely could be seen for other pathogenic parvoviruses as well, especially those that are capable of replicating in low or non-dividing cells, such as the copiparvovirus EqPV-H.

### Rat parvoviruses (RPV)

Rat parvoviruses have been recognized for over 50 years, with Kilham rat virus (KRV) being recovered from rat tumors and H-1 virus (H-1) from human tumor cell lines in 1959 and 1960, respectively [280, 281]. Rat parvovirus 1 (RPV1) was isolated from infected cell lines in 1998 and rat minute virus (RMV) was detected in naturally infected rats in 2002 [282, 283]. RPV1 infections are generally subclinical, and disease associated with experimental infections varies between serotypes of virus and age of the rat. Natural KRV infection of suckling rats was once reported to result in runting, ataxia, jaundice, and cerebellar hypoplasia, while infection of juvenile rats caused sudden death, scrotal cyanosis, and abdominal swelling [284].

### Other protoparvoviruses

Parvoviral DNA was isolated from archived tissues (2000–2013) of stranded southern sea otters and named sea otter parvovirus (SoPV, *Carnivore protoparvovirus 2*) [285]. PCR analysis of tissues of 69 otters revealed a 61% DNA prevalence with no change in prevalence rates over time, suggesting SoPV is endemic. DNA was most frequently detected in mesenteric lymph nodes, with much lower frequency in liver, lung, retropharyngeal lymph nodes, and spleen. The association of this virus with clinical disease in otters is unknown and experimental infection studies are unlikely due to its status as threatened subspecies.

Other protoparvovirus DNA has been discovered in dogs, wild wolves, foxes, pigs, bats, primates, and other hosts (Table 1). Canine bufavirus (CBuV, *Carnivore protoparvovirus 3*) was first detected in samples from three puppies with respiratory disease in Italy in 2011 [286]. The virus is a common component of the canine enteric virome, and viral DNA has been detected in feces of (1) diarrheic dogs in China [287, 288], (2) both healthy and sick dogs in Italy [289] and (3) wild wolves and foxes in Italy [290]. CBuV DNA was also detected in nasal and oropharyngeal swabs and enteric samples of young and adult domestic cats in Italy [291]. Fox parvovirus (FoPV, *Carnivore protoparvovirus 4*) DNA was detected in the fecal virome of foxes in the Netherlands in 2013 and in foxes in Croatia in 2016, but has not been associated with disease [292, 293]. Porcine bufavirus (PBuV, *Ungulate protoparvovirus 2*) DNA was detected in feces from domestic pigs with and without posterior paraplegia living at five affected, and one unaffected, farms in Hungary in 2016 [294]. The virus has been detected in diarrheic pigs in China and healthy pigs in the USA, although disease association is unknown [295–298]. California sea lion parvovirus DNA was detected in the mesenteric lymph node of a stranded, free-ranging California sea lion with disseminated granulomatous inflammation and necrotizing steatitis and vasculitis [299]. The clinical significance of this virus, however, remains undetermined as five additional mammalian viruses were also detected in the lymph node and in situ hybridization of multiple tissues was negative.

## Genus *Tetraparvovirus*

This genus was established in 2014 to recognize viruses discovered through metagenomics analyses (Fig. 2, Table 1). Human parvovirus 4 (PARV4) DNA was isolated from plasma of human patients with acute viral infection syndrome in 2005 [300], although its clinical significance remains undetermined. Porcine parvovirus 2 (PPV2, *Ungulate tetraparvovirus 3*) DNA was discovered in Myanmar in swine sera in 2001 [301], and more recently in China, Hungary, US, Germany, Japan, and Vietnam [302–307]. A high prevalence of PPV2 DNA was found in archived porcine serum and lung tissue samples collected between 1996 to 2013 in the US, and concurrent porcine circovirus type 2 (PCV2) DNA was seen in 14.3% of the pigs [186]. Since the prevalence of PPV2 DNA was significantly higher in tissues also containing PCV2 DNA, PPV2 may contribute to PCV-associated disease [186]. In situ PCR showed PPV2 DNA or RNA in lymphocytes in lungs, lymph nodes, and liver, of dead weaned pigs on PCV2-associated PMWS-negative and -positive farms in Hungary [306]. IHC co-staining for T and B lymphocytes, and macrophages, suggested that PPV2 may have a specific tropism for immature B lymphocytes and/or NK cells, but not T lymphocytes. Attempts to culture the virus in vitro have been unsuccessful and additional studies are needed to reveal any PPV2 disease association, as current evidence is weak (Table 2).

Bovine hokovirus 1 (BPARV4) DNA (species *Ungulate tetraparvovirus 1*) has been detected in samples of bovine spleen, and porcine parvovirus 3 (PPV3, *Ungulate tetraparvovirus 2,* formerly known as porcine hokovirus PPARV4) DNA was found in lymph nodes, liver, serum, and nasopharyngeal or fecal swabs of pigs [308]. In 2016, BPARV4 DNA was also detected in blood samples of yaks in China [309]. PPV3 DNA has been detected worldwide in healthy and sick pigs, but has not been linked to disease [185, 305, 308, 310]. DNA of the first tetraparvovirus of sheep, ovine hokovirus (OvPARV4, *Ungulate teraparvovirus 4*), was first isolated in 2011 from ovine liver and spleen samples [311]. Lastly, Eidolon helvum parvovirus 1 (BtPARV4, *Chiropteran tetraparvovirus 1*) was identified in the blood samples of flying fox bats of West Africa, though its clinical significance remains unclear [99].

## Genus *Chaphamaparvovirus*

Following the recent ICTV re-classification, this genus has been added under the new *Hamaparvovirinae* subfamily of *Parvoviridae* (Fig. 2, Table 1). The name chapparvovirus comes from the host groups in which its members were initially discovered (chiropteran, avian, and porcine), and currently contains 16 species, many recently discovered [20]. Similar to aveparvoviruses, chaphamaparvoviruses do not have a PLA_2_ domain in their VP proteins [20]. A recent analysis of MKPV identified several additional putative accessory proteins, namely p15, p10, and NS2/NP, whose functions are unknown (Fig. 3) [8].

### Mouse kidney parvovirus (MKPV)

MKPV belongs to the species *Rodent chaphamaparvovirus 1* [7, 8]. The discovery of MKPV helped resolve a 40-year-old mystery concerning the etiology of a condition in laboratory mice known as inclusion body nephritis (IBN), characterized by prominent, homogenous eosinophilic inclusions in the nucleus of renal tubular epithelial cells of immunodeficient mice [7, 312, 313]. The initial discovery of MKPV occurred after an increase in deaths of immunodeficient mice due to kidney failure [7]. Gross lesions included shrunken, pale kidneys, and these kidneys had tubular degermation and necrosis, tubular loss, interstitial fibrosis, and medullary papillary necrosis microscopically. The nuclei of numerous tubular epithelial cells contained large, amphophilic, intranuclear inclusions characteristic of a viral infection, but attempts to identify viral particles with EM were unsuccessful. RNA extraction and sequencing of kidney tissue from affected mice revealed two coding sequences with homology to the typical parvoviral NS and VP proteins [7]. To help fulfill some of Koch’s postulates, the authors demonstrated transmission of the virus through co-housing with virus-free mice. Importantly, co-housed mice had detectable MKPV DNA in the serum and urine after 50–80 days [7]. ISH of viral NA showed localization in tubular epithelial cells, and the abundance of the ISH signal correlated with the severity of disease further indicating disease association. Lastly, liquid chromatography-tandem mass spectrometry of affected kidneys revealed MKPV NS1 and VP1 peptides, indicating productive infection. RNA sequencing and mass spectrometry of affected kidney tissue also demonstrated expansion of activated macrophages and development of myofibroblasts in the kidney. Combined, these findings not only provided strong evidence of an etiology for IBN in mice, but also suggest a model of chronic kidney disease in humans (Table 2). Another study demonstrated viral NA in renal tubular epithelial cells, but not in liver or spleen, and combined with their findings of spliced MKPV RNA production in kidney cells only, suggest that that the kidney is the exclusive location for MKPV replication despite detection of DNA in other tissues [8]. While these reports focused on lesions in immunodeficient mice, other studies have shown that histologic lesions associated with MKPV infection of immunocompetent mice were similar, with lymphoplasmacytic tubulointerstitial nephritis with tubular denegation, although with rare intranuclear inclusions [11].

Similar viruses may be widespread in rodents, and a close relative (murine chapparvovirus (MuCPV, classified within the same species) was discovered in the feces of house mice in residential building of New York City [223]. A prevalence study of MKPV in laboratory mice using feces collected over a seven-month period from 78 biomedical research institutions found that 5.1% of mice tested positive by qPCR and in addition, 23.3% of pet mice from a pet store local to the authors in the US were also positive for MKPV [8]. These epidemiologic and metagenomic studies demonstrate that these viruses are widely distributed in laboratory, pet, and wild mouse populations around the world.

### Tilapia parvovirus (TiPV)

The partial genome of TiPV was first detected during a metagenomic analysis of crocodile feces in China [2]. After detecting a higher prevalence of this virus in feces of crocodiles fed tilapia versus those fed chicken, TiPV was identified in the intestines of tilapia [2]. TiPV was also detected during a severe mortality event in farmed adult tilapia in China in 2015, using a combination of EM, experimental infection, and ISH [6]. Clinical signs of affected fish included lethargy, anorexia, change in swim behavior, multifocal hemorrhage, and ocular lesions. Microscopically, fish had splenic necrosis, encephalitis, nephritis, hepatitis, and gill branchitis. EM revealed aggregates of non-enveloped capsids of ~ 30 nm in diameter in the cytoplasm and nucleus of cells of the heart, spleen, kidneys, brain, and gills. Both positive viral NA hybridization by ISH and viral DNA by PCR were detected at the highest levels in kidney and spleen, and experimental infection studies with purified TiPV from cell culture resulted in similar lesions compared to naturally occurring disease, providing strong evidence of pathogenicity [6] (Table 2). Phylogenetic analysis revealed that TiPV NS1 protein aa sequence was most closely related to porcine parvovirus 7 (PPV7) of the same genus. TiPV is clearly a pathogenic virus in tilapia, an important economic species for aquaculture worldwide, and is the first parvovirus confirmed to infect fish. More recently, TiPV was co-detected with Tilapia lake virus in a natural disease outbreak of farmed tilapia in Thailand, further emphasizing the significance of this pathogen [314].

### Other chaphamaparvoviruses

PPV7 (*Ungulate chaphamaparvovirus 1)* was first discovered in pooled rectal swabs of adult pigs in the US in 2016, and has subsequently been detected in China, Korea, Poland, Sweden, and Brazil [315–320]. Some studies suggest that this virus has a more rapid evolutionary rate compared to other PPV genotypes [321]. However, a direct link between infection and clinical disease has not been established.

Two novel parvoviruses were detected in stool samples from dogs suffering from an infectious diarrhea outbreak in the US in 2017 [322]. These closely related viruses, called Cachavirus (CachaV)1 and − 2 (*Carnivore chaphamaparvovirus 1*) were subsequently demonstrated in the stool of both diarrheic and healthy dogs in China and Italy, suggesting that those are not a direct cause of the disease [323, 324]. CachaV DNA was also detected in the feces of two diarrheic cats in China, but there was no statistically significant association between the presence of the virus and clinical signs [325].

Identified avian chaphamaparvoviruses include turkey parvovirus 2 (TPV2, *Galliform chaphamaparvovirus 1)* detected in feces of a 1-year-old turkey with diarrhea in Hungary [326], and chicken chapparvovirus 2 (ChikPV2, *Galliform chaphamaparvovirus 2*) identified in feces of chickens that were either sick with malabsorption syndrome or healthy [327]. There were no statistically significant differences in the distribution of viral sequence reads identified in either healthy or sick birds. Discoveries of chapparvoviruses in other bird species include parakeets [328], peafowls [329], and red-crowned cranes [127]. A study of Canadian ducks detected duck-associated chapparvovirus (DAC) in paired oropharyngeal and cloacal swabs of apparently healthy ducks and *Galliform chaphamaparvovirus 3* in a gull [330].

Many chapparvovirus sequences have been detected in metagenomic studies of bat viromes, including *Desmodus rotundus* chapparvovirus (DrPV-1, *Chiropteran chaphamaparvovirus 1*), which was identified in kidney tissue from vampire bats in Brazil [331]. Tasmanian devil-associated chapparvovirus 1, 2, and 6 (TdChPV, *Dasyurid chaphamaparvovirus 1–3*) were identified during a metagenomic analysis of the Tasmanian devil virome in 2019 [332]. Capuchin kidney parvovirus (CKPV, *Primate chaphamaparvovirus 1*) was detected in the kidney of a wild capuchin monkey and had a high level of identity with MKPV [8]. A novel chaphamaparvovirus called fechavirus (FChPV, *Carnivore chaphamaparvovirus 2*) was identified in six cats during an outbreak of vomiting and diarrhea in a system of shelters in Canada in the same study that also identified three novel bocaparvoviruses [151]. Psittacara leucophthalmus chapparvovirus (PlChPV, *Psittacine chaphamaparvovirus 1*) was identified in fecal specimens of wild birds in Brazil [328]. Bearded dragon chaphamaparvovirus (BDchPV) was identified along with a novel circovirus during a meta-transcriptomic investigation of a mass mortality and morbidity event in a bearded dragon colony associated with extensive proliferation of the respiratory epithelium [333]. Lastly, a rat parvovirus 2 (RPV2, *Rodent chaphamaparvovirus 2*) has been identified in a metagenomics study of adult rats in China in 2016 [334].

## Animal parvoviruses as therapeutics for human diseases

As mentioned previously, human AAVs have received high interest because of their use as powerful tools for gene therapy in humans, since the first use of an AAV vector for gene delivery in 1984. After a long period of development, a number of recombinant (r)AAVs have recently been approved for use in humans. Alipogene tiparvovec (Glybera), an AAV-based gene therapy treatment for lipase deficiency, was the first approved rAAV gene therapy for use in humans and was approved by the European Medicines Agency in 2012 [335]. Voretigene neparvovec-rzyl (Luxturna), an rAAV therapy to treat *RPE65* mutation-associated inherited retinal disease was approved in the US in 2017 [336]. However, a challenge with using human AAVs for gene therapy is the presence of pre-existing anti-AAV capsid antibodies in humans that block the viruses [337]. Some AAVs from other hosts may also be affected by immunity in humans. For example, AAV8 from Rhesus macaque is blocked by neutralizing antibodies in 10–40% of people in many populations, depending on the geographical region [337, 338]. To reduce the likelihood of pre-existing immunity in humans, the use of AAV capsids from other animals which do not normally infect humans and induce pre-existing immunity are being explored. For example, porcine-derived rAAVs successfully transduce mouse tissues with a similar efficiency as traditional AAVs, and were not neutralized by pooled human immunoglobulin G [337, 339]. Similarly, the bat AAV strain 10HB has been proposed as a possible platform for carrying AAV2 vector genomes given the lack of anti-viral antibodies in human sera [221, 222].

Other parvoviruses have also been proposed for human gene therapy. For example, bocaviruses package an approximately 10% larger genome than AAVs [340]. Gorilla bocavirus 1 (GBoV1) from gorillas [341], has been suggested as a gene therapy delivery vector instead of HBoV1 [342]. HBoV1, which is associated with respiratory disease in humans, is valued as a gene therapy vector because of its specific tropism for the apical side of polarized human airway epithelial cells, thus, providing a therapeutic option for diseases such as cystic fibrosis [33, 343]. GBoV1, like HBoV1, is also able to infect the apical side of polarized human airway epithelial cells and appears less susceptible to neutralization by human immunoglobulins [340].

Autonomous animal parvoviruses are also being developed as human anti-cancer agents and the role of parvoviruses in oncolytic therapy has been recently reviewed elsewhere [344]. Rodent parvoviruses, and in particular the non-pathogenic H1, have showed promising results as oncolytic viruses due to their high safety profile and natural oncotropism. H1 infection of cancer cells results in cell lysis and secondary stimulation of the immune response through the release of danger-associated molecular patterns (DAMPs), viral pathogen-associated molecular patterns (PAMPs), and tumor-associated antigens [345].

## Conclusion

The number of parvoviruses and parvoviral sequences has increased dramatically in recent years due to improvements in viral discovery approaches and studies in domestic animals and many wild species, including some that are critically endangered. A better understanding of these parvovirus infections, and identification of those with pathogenic potential, will help to explain the etiology of many diseases, and might increase the chances of rescuing those animal species that are endangered. Lastly, as shown by the recent emergence and widespread impact of SARS-CoV-2 as well as by the pandemic emergence of canine parvovirus in the 1970s, the significance of any newly discovered virus is not always obvious at first. This review clearly shows that we can expect the same for parvoviruses, since new pathogenic viruses continue to be identified around 100 years after the first parvovirus diseases were reported.

## Data Availability

Not applicable.

## Abbreviations

aa: Amino acid
AAV: Adeno-associated virus
AD: Aleutian disease
CSF: Cerebrospinal fluid
DDR: DNA damage response
EM: Electron microscopy
ICVT: International Committee on Taxonomy of Viruses
IHC: Immunohistochemistry
ISH: In situ hybridization
ITR: Inverted terminal repeat
NA: Nucleic acid
NGS: Next generation sequencing
NS: Nonstructural protein
ORF: Open reading frame
PLA2: Phospholipase A2
rAAV: Recombinant adeno-associated virus
*Rep*: Replication
RSS: Runting and stunting syndrome
SBDS: Short beak and dwarfism syndrome
SF3: Super family 3
ss: Single stranded
US: United States of America
VP: Viral protein
